# The course of specialization in public health in Rio de Janeiro, Brazil, from 1926 to 2006: lessons and challenges

**DOI:** 10.1186/1478-4491-8-4

**Published:** 2010-03-05

**Authors:** Monireh Obbadi

**Affiliations:** 1 National School of Public Health of Brazil (ENSP), Rua Leopoldo Bulhões 1480, Manguinhos, 21041- 210 Rio de Janeiro - RJ, Brazil

## Abstract

**Background:**

Public health, as a field of knowledge, depends on its professionals. Their education and training, therefore, is considered to be an important factor for the quality of health services. In Brazil, the Course of Specialization in Public Health of the National School of Public Health is one of the oldest in the country. The course has existed for over 80 years, during which it has had an eventful history, with modifications in its organization, interruptions in its delivery, threats to its survival and changes in the institutions hosting it, reflecting the wider transformation in Brazilian society and public life over that period.

**Methods:**

In this article we analyse this course via its history, disciplines, organization and characteristics of the student body.

**Results:**

Insights were gained into the advancement of public health in Brazil and the progress of education for professionals in this field was highlighted. The course has formed nearly 2000 specialists in public health.

**Conclusions:**

An analysis of the course's history provides valuable lessons for other schools of public health trying to train professionals in developing countries.

## Background

In 2006 the Course of Specialization in Public Health currently administered by the National School of Public Health of Brazil (ENSP) completed 80 years of existence. During this period, this course has had an eventful history, with modifications in its organization, interruptions in its delivery, threats to its survival and changes in the institutions hosting it.

An analysis of the history of the course provides an insight into the development of public health education in Brazil, reflecting the wider transformation in Brazilian society and public life during this period. The records show how external events have impacted this development, in particular the establishment of the National School of Public Health in 1954, which took over the administration of the course. Another important event - with the promulgation of the new Brazilian constitution of 1988 - was the advancement of the health reform movement which culminated in the creation of the Brazilian National Health System (SUS). Other external factors were the changes in government and ensuing reorganizations of public services, in particular those associated with the military revolution of 1964 [1].

## Methods

In this article we analyse the course via its history, disciplines, organization and characteristics of the student body in order to gain an insight into the development of public health in Brazil and to highlight the progress of education for professionals in this field.

## Results & Discussion

The early decades of the twentieth century were a time of increased interest in public health in Brazil. This was due to both the greater realization of the microbial risk of infection and a belief that improved public health was a sign of progress and modernization of the country. Within the Americas, the creation of the Pan-American Sanitary Bureau in 1902 and the Rockefeller Foundation in 1913 gave political, technical and financial support to the burgeoning health reform movements in the New World [2] while the Flexner report of 1910 [3] led to the reform of medical education. In Brazil, this increased interest was reflected in the creation, by decree in 1919, of the National Department of Public Health (DNSP) and the Brazilian Society for Hygiene in 1923 [4].

It was also during this period that in 1925 the Special Course in Hygiene and Public Health, the forerunner of the present Course for Specialization in Public Health, was created. The Faculty of Medicine of Rio de Janeiro together with the Oswaldo Cruz Institute, situated in the same city, initially administered the course.

### The Early Years

There is little information available on the initial years of the course. Its creation was a consequence of the sanitary reform movement of 1920-23 and the reform of medical education, which occurred in Brazil following a plan elaborated in 1925.

Carlos Chagas, the renowned Brazilian research scientist and director of the Oswaldo Cruz Institute wished to collaborate more closely with the Faculty of Medicine. He wanted to bring hygiene and clinical problems related to rural diseases into medical practice, thereby creating a new career for health professionals. At that time his idea was controversial within the medical faculty, as it involved both the creation of the new chair of tropical diseases as well as bringing outside lecturers from the Oswaldo Cruz Institute to teach the new course, the Special Course in Hygiene and Public Health [5].

The Rockefeller Foundation was contacted for assistance with the course. Although there is no record of direct financial assistance, the foundation financed the visit of two professors from the Johns Hopkins School of Hygiene and Public Health to assist with the teaching of the disciplines of epidemiology and hygiene management. This assistance not only helped to strengthen the course but also broadened the curriculum from the original French and German base [6].

In 1930, the DNSP was given a new name, the National Department of Health (DNS), as part of a reorganization of public services. The DNS assumed responsibility for the course, which from 1931 became known as the Course on Hygiene and Public Health. In 1940 and 1941, changes were made to the organization of the course. Barros Barreto [7], the director general of the National Department of Health, reporting on these changes to the then Minister of Health, Gustavo Capanema, demonstrated his concerns for the critical situation of public health in Brazil. He reported that the Course on Hygiene and Public Health during its first 15 years of existence had only formed eight cohorts of medical hygienists. He lamented the interruption of the course in 1937 and boldly demonstrated his preoccupation with the then current state of public health education and preparation of the professionals in this field. He focused on the necessity of the government to support, reinforce and stabilize the public health profession and questioned the lack of attention given by the government to the preparation of human resources for the public health profession.

Barros Barreto stressed that the government should give more importance and attention to the formation of specialists of public health in preference to other medical specialties. He believed the students of the Course on Hygiene and Public Health were an important element of action to deal with prevention and combat of disease and ill health. On the other hand he also criticized the lack of time given to, and poor quality of the public health disciplines taught at, the faculties of medicines. As a result of his report, further improvements were made to the organization of the course.

### Disciplines

An analysis of the evolution in the choice of disciplines administered by the course mirrors the changes that occurred in public health education during this period. The course originally was divided into theoretical and practical classes. In the mornings there were classes in chemistry, nutrition, bacteriology and immunology among other subjects. The afternoons were dedicated exclusively to practical lessons, which included clinical demonstrations and self-study.

With the changes that occurred in 1931 the disciplines of the course included the following: health statistics; urban and rural sanitation; epidemiology and prophylaxis of acute contagious diseases; epidemiology and prophylaxis of other transmissible diseases especially rural endemics diseases; leprosy; venereal diseases and cancer; physiology applied to hygiene; food and industrial hygiene; infantile hygiene, hygiene management and organization. The additional disciplines of microbiology and parasitology applied to public health were dispensed for students who were graduates from the official faculties of medicine or who held the diploma of the famous application course of the Oswaldo Cruz Institute.

The choice of the disciplines demonstrated the preoccupation of the authorities during this period with promoting cure and with individual care. At this time, health was more concerned with treating the individual rather than preventing disease [8]. The curriculum was similar to the first schools of public health established in North America, such as the Johns Hopkins School of Hygiene and Public Health, the Harvard School of Public Health, the School of Hygiene at the University of Toronto, the DeLamar Institute of Public Health at Columbia University and the Department of Public Health at Yale University. Their curricula in the 1920s and 1930s were also heavily weighted towards the laboratory sciences, epidemiology and statistics [9].

With the reforms of the 1940s the disciplines of mental hygiene, nutrition and diagnosis of communicable diseases were also added. Barros Barreto [7] had criticized the fact that more time was given to the disciplines of microbiology and parasitology that were administered by the Oswaldo Cruz Institute then was given to the other 10 disciplines combined. He also called for a closer link between the course and the federal health services. His call was similar to an earlier effort by the Rockefeller Foundation to establish programs of field training in North American Schools which lead the John Hopkins School of Hygiene and Public Health to create the Eastern Health District consisting of a study population of 100 000 people [9].

With the creation of the National School of Public Health, further changes occurred. In 1959, the discipline of sociology as applied to medicine and public health was added. The objective was to approximate the medical activities with the community and to make the students more aware of national problems and changes in the social context. Specifically the course intended to provide solid facts for the sociological analysis of contemporary Brazilian society and to make the health professionals understand better the need to make plans which took into account all the relevant considerations and necessities of the population.

With the democratic reforms, which occurred in Brazil during the late 1970s, a change also occurred in the nature of the disciplines of the course. There was a general move away from biology and hygiene and towards administration, planning, management and human resources. Disciplines such as social sciences, group dynamics, ecology, introduction to the theory of knowledge, problems of Brazilian health and biostatistics were prominent. This was also a response to efforts from the Pan-American Health Organization (PAHO) to bridge the gap between academic public health institutions in the continent and the necessities of the public health work force through the Program for Strategic Preparation of Health Personnel (PPREPS/PAHO) and the Program for the Internalization of Actions in Health and Sanitation (PIASS) [10].

These modifications reflected the change in vision of public health education, from a preoccupation with individual health to that of concern about community health. They were also a consequence of the arrival at the National School of Public Health of a group of professors from the university of Campinas, lead by Sergio Arouca, who became one of the leaders of the health reform movement in Brazil and a leading architect of the national health system established by the Brazilian constitution of 1988. These professors brought with them new competencies and ideas as well as contacts with federal funding agencies such as FINEP. Their new vision of public health was reflected in the reformulation of the curriculum [11].

The structure of the course was also changing. In the mid 1980's the disciplines were rearranged into modules, which in turn were replaced by four thematic areas: the study of social sciences as applied to health; epidemiology; environment and public health; and administration and planning of health services. From 2000 the course was based on three pillars: health promotion, health surveillance and health research. The principal themes were public policies, health and society, epidemiology, statistics, demography, and management and planning of health services.

### Characteristics of the student body

The number of candidates for the course, the number of students who passed the selective process and were enrolled and the number of students receiving the diploma are shown in Table 1, for each decade of the history of the course. The changes in student numbers were a consequence of various factors including re-organizations of the course, availability of places, varying interest in the field of public health as a career, and the availability of scholarships.

**Table 1:** Number of students of the Course of Specialization in Public Health (1926-2006)

Period	No. of candidates	No. of students enrolled	No. of students receiving diploma
1926 - 1939	----	-----	159

19 40 - 1949	321	208	188

1950 - 1959	230	168	94

1960 - 1969	609	349	289

1970 - 1979	1022	487	433

1980 - 1989	1667	330	309

1990 - 1999	877	250	223

2000 - 2006	1428	220	178

**Totals**	**6154**	**2012**	**1873**

Source: Archives of the Academic Registrar of the National School of Public Health, Rio de Janeiro.

Originally, only students with a diploma in medicine could be candidates for the course. The course was destined exclusively for physicians who upon the conclusion of their studies were expected to occupy the function of specialized public health inspectors in the federal offices of the National Department of Health (DNS) or in the state offices.

In the 1960s, with the establishment of the National School of Public Health, the course was radically changed. It became a basic course in public health for professionals of different areas such as nurses, engineers, pharmacists and veterinarians, as well as for physicians. A specific program for each professional area was developed within the course.

This change in the composition of professionals taking the course also changed the gender balance. Initially, all the physicians participating in the course were male. The first female participated and concluded the course in 1938. During the 1940s a further five women registered for the course. However with the opening of the course to other professions, female participation greatly increased and currently female students are in the majority. During the 15-year period from 1992-2006 (inclusive), 74.5% of the students who completed the course were female.

During the 1980s, agreements were signed with the Superintendence of Campaigns (SUCAM) and the Foundation for Special Services in Public Health (FSESP), in order for professionals from the health services to participate in the course. With the municipalization of health services, which were intensified following the promulgation of the new Brazilian constitution of 1988, an increase of applicants were seen from municipal health services, particularly from the state of Rio de Janeiro. During the period 1992-1999, 36.1% of the students who completed the course were from municipal health services, 34.8% came from the state and federal sectors, and the remaining students either came from the private sector or were not employed or were from other countries.

Nunes, analyzing these facts, concluded that the increase in candidates could be due to two factors [11]. Firstly, the course was increasingly being targeted to professionals from multiple disciplines. Second, advocacy was undertaken by the National School of Public Health, directed at the other government institutions, so that the diploma of the course was recognized as a valid title for public health professionals and consequently brought financial benefit for those who completed the course.

### Selection criteria

Initially, the selection process was based on written and oral tests and the result was published in the official register (Diário Oficial). Later, the selection criteria became more organized with written tests in mathematics, chemistry, physics, general biology and haematology as well as an oral test. A board of examiners composed of three professors judged the tests. The final score of the candidate was the result of the average score given by the three professors.

In order to register, candidates had to present the following documents: a) proof of identity; b) proof of completion of, or exemption from, military service; c) certification of vaccination against smallpox and typhoid; d) certification of physical and mental health.

With the establishment of ENSP, changes to the course were introduced gradually. English was introduced to the selection test and the oral exam eliminated. The selection criteria were also changed with a single test of general knowledge related to each area of the course. The examining board had members from the different areas of health.

Currently candidates are selected based on a two-stage process. The first stage consists of a written exam, which is eliminatory. Candidates who pass the first stage are classified after an analysis of their curriculum vitae and an interview.

### Organization of the Course

Originally, the duration of the course was twelve months of full-time study starting in January. The teachers and their assistants belonged to the ministry of education and health and were entitled to a special bonus payment. The majority of students received scholarships, with some places reserved for physicians from other states. In 1938 the course was also offered for the first time in Recife in the North East of Brazil.

In the 1940s, twelve professors with their assistants ministered the disciplines of the course. The disciplines were divided into four periods and at the end of each period there were written, practical and oral tests. The course was organized with practical and theoretical classes, visits and even excursions. From the content of the disciplines, the preoccupation of the coordinators to provide a broad vision of public health can be observed.

The director general of the National Department of Health (DNS) approved the program of the course. The students who obtained the certificate of the course in public health and who wished to take another course in the area of health had the privilege of not needing to participate in a further selection process.

Students who failed any discipline could not continue with the course. However, they had a chance to repeat the discipline the following year. If they failed the re-sit exam they were expelled from the course.

With the establishment of ENSP, full-time ministration of the disciplines continued, sub-divided into four periods of between 140 to 290 hours of classes. During this time an increase in the number of candidates from other states of Brazil was noted. This was one of the reasons for the creation of the decentralized courses in public health in nearly all the states of Brazil and which were coordinated by ENSP. These courses stimulated the creation of regional nuclei for the formation of human resources in health, some of which have in turn become independent schools of public health.

In the mid 1970s, two courses in public health were offered: a basic course destined for professionals working directly with the public; and a specialized course for those who completed the former and wished to become specialists in the area of public health. In 1982, the two courses were combined into a single Course of Specialization in Public Health.

During the 1980s, a number of key events, including the promulgation of the new constitution and the creation of the New Republic, the 8^th ^National Health Conference and the creation of the National Health System (SUS) had important effects on the National School of Public health, which went through a process of significant transformation. These transformations reflected on the methodological organization, the content and the candidates of it courses.

In order to meet the needs of students who originated from the health services, the course ceased to be offered on a full time basis from 2001. Currently students are only required to attend classes for two days per week. The course provides 490 hours of theoretical class work and students are still required to produce a monograph equivalent to 200 hours work.

## Conclusion

The Course of Specialization in Public Health has survived for over 80 years. During this period, Masters and doctorate courses in public health have been created at ENSP, as well as medical residencies in this specialty. Other institutions, such as the University of São Paulo and the Federal Universities of Bahia and Rio Grande do Sul, have developed their own prestigious programs of public health education. According to Buss [12], Brazil now has one of the greatest concentrations of public health training programs in the world. One might have thought that with such competition, together with the establishment of the decentralized public health courses, the Course of Specialization in Public Health would no longer prove to be attractive.

In fact the opposite has occurred. The present decade has seen an unprecedented demand for places on this course. Part of this demand can be considered as accompanying the general trend of increasing interest in higher education, and the fact that undergraduate (first degree) courses in public health in Brazil have only recently begun to function.

Part of the credit for the continuing popularity of the course, however, has been its ability to adapt to the demands and needs of public health in Brazil. During its 80 years of history, the course has formed nearly 2000 specialists in Public Health, including many who have gone on to occupy important positions in federal and state public health administration.

The history of the course provides valuable lessons for other schools of public health trying to train public health professionals in developing countries. The course has changed its focus, from its original aim of training medical physicians for important posts in the public health bureaucracy to preparing a wide range of professionals for future careers in the different fields of public health. The course has also continuously faced the challenge of avoiding the "divorce between theory and practice" that has often characterised public health education in North America [9].

Pedagogically, the course has changed from an emphasis on practical training with a focus on biology, to critical thinking with a focus on sociological studies. It has had to straddle the paradox of providing a specialized course for a generalized training while also ensuring that the competencies it is teaching remain valid for the modern public health professional. The course has achieved these aims by constantly changing its organization, redefining the selection criteria of its students and updating the choice of the disciplines it administers.

## Competing interests

The author declares that she has no competing interests.
